# Dual blockade of EGFR and VEGFR pathways: Results from a pilot study evaluating apatinib plus gefitinib as a first‐line treatment for advanced EGFR‐mutant non‐small cell lung cancer

**DOI:** 10.1002/ctm2.33

**Published:** 2020-06-04

**Authors:** Zhonghan Zhang, Yang Zhang, Fan Luo, Yuxiang Ma, Wenfeng Fang, Jing Zhan, Su Li, Yunpeng Yang, Yuanyuan Zhao, Shaodong Hong, Ting Zhou, Yaxiong Zhang, Shen Zhao, Yan Huang, Hongyun Zhao, Li Zhang

**Affiliations:** ^1^ Department of Medical Oncology State Key Laboratory of Oncology in South China Collaborative Innovation Center for Cancer Medicine Sun Yat‐sen University Cancer Center Guangzhou Guangdong P. R. China; ^2^ Department of Clinical Research State Key Laboratory of Oncology in South China Collaborative Innovation Center for Cancer Medicine Sun Yat‐sen University Cancer Center Guangzhou Guangdong P. R. China

**Keywords:** antiangiogenic therapy, Apatinib, EGFR‐TKIs, Gefitinib, NSCLC

## Abstract

**Background:**

Dual blockade of both EGFR and VEGFR pathways in EGFR‐mutant NSCLC have shown enhanced antitumor efficacy versus EGFR‐TKIs alone. Apatinib is an orally effective VEGFR‐2 tyrosine kinase inhibitor (TKI). This pilot study aims to evaluate the tolerability, pharmacokinetic profile, and antitumor activity of apatinib plus gefitinib as a therapy for EGFR‐mutant advanced NSCLC.

**Methods:**

Advanced non‐squamous NSCLC participants harbored with the EGFR 19 deletion or the 21 L858R point mutation were included. There were two cohorts: Cohort A: apatinib 500 mg + gefitinib 250 mg. Cohort B: apatinib 250 mg + gefitinib 250 mg. The primary endpoint was safety profile. Other endpoints consisted of PK analysis, objective response rate (ORR), and progression‐free survival (PFS). Exploratory analysis was conducted using next‐generation sequencing of plasma circulating‐tumor DNA.

**Results:**

Between July 2016 and April 2017, 13 of NSCLC patients were recruited. Six patients were pooled in Cohort A, while seven patients were in Cohort B. Adverse events (AEs) were tolerable (mostly grade 1–2) and the treatment‐related AEs were similar in both cohorts: rash (100% vs 71.4%), diarrhea (66.7% vs 71.4%), hypertension (66.7% vs 71.4%), proteinuria (66.7% vs 42.9%), and hand‐foot skin reaction (33.3% vs 28.6%). The area under plasma concentration‐time curve for the steady state of apatinib was 2864.73 ± 2605.54 ng mL^–1 ^h^–1^ in Cohort A and 2445.09 ± 1550.89 ng mL^–1 ^h^–1^ in Cohort B. Of the 11 patients evaluable for efficacy, Cohort A achieved an ORR of 80.0% and reached a median PFS of 19.2 months, while it was 83.3% and 13.4 months in Cohort B. Patients without a concomitant mutation at baseline had a prolonged PFS tendency (20.99 months v 13.21 months, *P *= .0624). The EGFR‐T790M mutation remained the dominant resistance mechanism.

**Conclusion:**

Apatinib (500 mg) plus gefitinib (250 mg) showed a tolerable safety profile and encouraging antitumor activity for advanced EGFR‐mutant NSCLC in the first‐line setting. Phase III trials of apatinib (500 mg) plus gefitinib (250 mg) are warranted.

**Trial registration:**

Clinicaltrials.gov, NCT02824458, date of registration June 23, 2016.

**Abbreviations**
AEs: adverse events
AUC: area under the plasma concentration‐time curve
AUCss: area under the plasma concentration‐time curve for steady state
BOR: Best overall response
CLss: steady state clearance rate
C_max_: maximum plasma concentration
CR: complete response
Css: steady state concentration
ct‐DNA: circulating tumor DNA
DCR: disease control rate
ECOG: Eastern Cooperative Oncology Group
EGFR‐TKIs: epidermal growth factor receptor tyrosine kinase inhibitors
NGS: next generation sequencing
NMPA: National Medical Products Administration
NSCLC: non‐small cell lung cancer
ORR: objective response rate
OS: overall survival
PD: progressive disease
PFS: progression‐free survival
PK: pharmacokinetic
PR: partial response
RECIST: Response Evaluation Criteria in Solid Tumors
SAEs: severe adverse events
SD: stable disease
T_1/2_: elimination half‐life
T_max_: time to reach maximum plasma concentration
Vdss: steady state volume of distribution
VEGFR: vascular endothelial growth factor receptor

## 1 BACKGROUND

Non–small cell lung cancer (NSCLC) is by far the leading cause of cancer‐related deaths all over the world.^1^, ^2^ Approximately 30‐40% of NSCLCs are found to be locally advanced or metastatic at the time of their initial diagnosis.^3^ Moreover, an epidermal growth factor receptor (EGFR) mutation is a driver gene of importance in NSCLC^4^ and its frequency is higher in Asian (about 30‐40%)^5^, ^6^ than in European and American populations (about 22%).^7^

Gefitinib was the first EGFR tyrosine kinase inhibitor (TKIs) approved for treating NSCLC patients who had an activating EGFR mutation. However, despite its remarkable anti‐tumor response and the prolonged progression‐free survival(PFS) rates,^8^, ^9^, ^10^, ^11^, ^12^, ^13^ resistance inevitably develops within most patients at around 10 months. Thus, it has become an urgent challenge to explore novel strategies for delaying the resistance of EGFR‐TKIs monotherapy.

Recently, antiangiogenic agents were highlighted as part of a combination therapy for the option of advanced NSCLC.^14^, ^15^ The phase II JO25567 study revealed that bevacizumab plus erlotinib may prolong PFS in NSCLC patients that presents the activating EGFR gene mutation, compared with erlotinib alone.^16^ Subsequently, a series of studies began investigating the potential of an anti‐EGFR combined with an anti‐VEGFR.^17^, ^18^, ^19^, ^20^, ^21^

Apatinib, an oral TKI that reporting to target the vascular endothelial growth factor receptor (VEGFR)‐2 selectively, has shown antitumor activity in several types of malignancies.^22^, ^23^, ^24^ In 2014, it was approved by the Chinese National Medical Products Administration (NMPA) as a third‐line monotherapy for advanced gastric cancer patients, in which it improved PFS and OS significantly with an endurable toxicity spectrum. A phase II trial also demonstrated that apatinib possessed a potential clinical activity without paralleling additional toxicity in advanced NSCLC cases who suffered from at least two front‐line therapies including the standard of care by EGFR‐TKIs.^25^

Therefore, we conducted this pilot study to assess the safety, pharmacokinetic (PK) profile as well as therapeutic efficacy of apatinib plus gefitinib in non‐squamous EGFR‐mutant NSCLC patients. We also performed next generation sequencing (NGS) of plasma circulating tumor DNA (ct‐DNA) to better understand the resistance development to this combination therapy.

## 2 METHODS

### 2.1 Patients

This pilot study was conducted between July 2016 and April 2017. The inclusion criteria were: (a) age between 18–75; (b) with recurrent or metastatic non‐squamous NSCLC; (c) a clinically diagnosed and activate EGFR mutation (19 deletion or 21 L858R mutation); (d) a performance status between 0 to 1 with Eastern Cooperative Oncology Group (ECOG) Scale, and (e) have not less than one measurable lesion at baseline by imaging examinations, based on the version 1.1 of Response Evaluation Criteria in Solid Tumors (RECIST 1.1) (see Supporting Information). Detection of an EGFR mutation basing on clinical samples was required for all enrolled patients (see Supporting Information). Screening of patient was completed at Sun Yat‐sen University Cancer Center (SYSUCC) according to the protocol and amendment approved by SYSUCC independent ethics’ committee (approved ID: 5010‐2016‐03‐01). This study followed the Good Clinical Practice Guidelines and the principles of the Declaration of Helsinki. Written informed consent form (ICF) was collected. This trial is part of the ACTIVE study and has a registration at website of www.clinicaltrials.gov (NCT02824458).^26^

### 2.2 Study design and drug administration

The safety and PK profile of apatinib plus gefitinib was the primary objective. Other endpoints included objective response rate (ORR) and disease control rate (DCR), progression‐free survival (PFS), and overall survival (OS). There were two cohorts: Cohort A: apatinib 500 mg + gefitinib 250 mg that was taken at the same time every day on an empty stomach for each 4‐week cycle. Cohort B: apatinib 250 mg + gefitinib 250 mg that was taken at the same time every day on an empty stomach for each 4‐week cycle. Combination therapy was continued until the status of progression disease, unbearable toxicity, withdrawal of informed consent form or discontinuation that was at the discretion of the principal investigator (see Supporting Information).

### 2.3 Study assessments

Adverse events (AEs) in line with the version 4.0 of National Cancer Institute's Common Terminology Criteria for Adverse Events (CTCAE 4.0) were evaluated by the investigators. AEs were strictly recorded from the timepoint that ICF was obtained to 28 days after the finish of study. Imaging examinations was conducted before treatment, at the finish of the first follow‐up, and every other two follow‐ups afterward by taking the same examinations. Response of tumor was assessed based on the RECIST 1.1 guidelines. Primary assessment for antitumor response: (a) ORR, defining as the proportion of participants that achieved a complete response (CR) or a partial response (PR); (b) disease control rate (DCR), defining as the proportion of participants reached a status of CR or PR or stable disease (SD). After 4 weeks, best overall response (BOR) was confirmed.

### 2.4 Pharmacokinetic analyses

In the two cohorts, PK assessment consisted of a single‐dose‐administration on Cycle 1, Day 1's (C1, D1) test and a continuous‐dose‐administration on Cycle 1, Day 15′s (C1, D15) test. For detailed blood collection administration, see the supplementary materials. Upon collection, the samples were centrifuged and plasma frozen at a refrigeration of −80 ± 5°C until further analysis. Apatinib and gefitinib plasma concentrations were measured using a confirmed liquid chromatography–tandem mass spectrometry method. PK parameters, including maximum plasma concentration (*C*_max_), area under the plasma concentration‐time curve (AUC), time to reach maximum plasma concentration (*T*_max_), elimination half‐life (*T*_1/2_), steady state concentration (Css), area under the plasma concentration‐time curve for steady state (AUCss), steady state volume of distribution (Vdss), and steady state clearance rate (CLss) were calculated using non‐compartmental methods with Winnonlin 7 software. The AUC was estimated by using the linear trapezoidal rule method and *T*_1/2_ was determined by linear regression of the terminal slope of the logarithmic plasma concentration‐time profile. Descriptive statistics were measured for the PK parameters using mean with its corresponding standard deviation (SD).

### 2.5 NGS of plasma ct‐DNA

NGS for patients’ plasma ct‐DNA was collected at baseline, best of response and after progression disease (PD) using a ct‐DNA panel of 15 critical genes, including point mutation, small fragment deletion and insertion, copy number changes, and known types of fusion genes. The median depth of reliable mutations detected within this panel was 5000X‐10000X. See Supporting Information for more (Tables S1 and S2).

### 2.6 Statistical analyses

Patients who taken at least one dosage of apatinib and gefitinib were pooled in the intention‐to‐treat (ITT) analysis. Patients in the ITT analysis who had complete safety records were pooled in the safety set (SS). The qualitative and quantitative data were summarized with the descriptive statistics. Efficacy was also assessed in the ITT analysis. The Kaplan‐Meier method described PFS and OS. Survival distribution differences were evaluated by the log‐rank test. The 95% CIs were calculated for PFS and OS to assess treatment efficacy. Fisher's exact test compared the response rate in the various subgroups. Two‐sided tests were applied. A *P*‐value < .05 was deemed to be statistically significant. All statistical analyses and graphs were made by the Statistical Package for Social Sciences (SPSS) 23.0 and GraphPad Prism 7.

## 3 RESULTS

### 3.1 Patients

**FIGURE 1:**
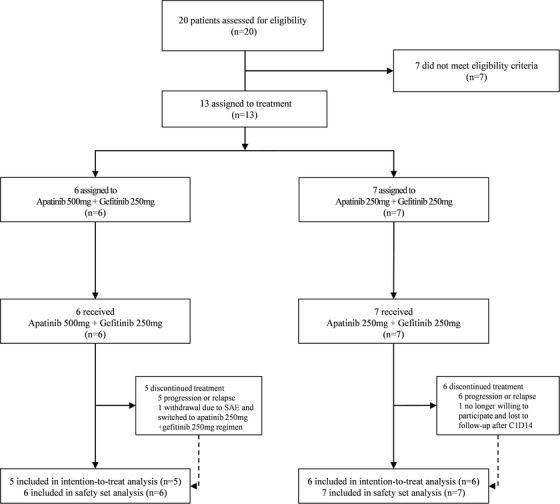
Trial profile

Thirteen patients were finally recruited in this study. Demographics and detailed characteristics are listed in Table 1 and Table S10. The majority of the patients (61.5%) had the EGFR exon 21 L858R point mutation. EGFR mutations were detected by PCR sequencing or ARMS method based on the tissue results of the 12 patients (the detectable lower limit of mutation abundance = 1%), while in one patient the mutation was determined by the NGS method (the allele frequency cutoff also = 1%) using a peripheral blood sample (Tables S3 and S4). One patient withdrew due to a severe adverse event (SAE), and one patient was unwilling to participate and follow‐up was lost after cycle 1, day 14 of the treatment. Only 11 patients were pooled in the ITT analysis. Figure 1 summarized the trial profile.

**TABLE 1:** Baseline patient demographics and clinical characteristics

Apatinib Dose Cohort	500 mg		250 mg	
Characteristic	No.	Percentage	No.	Percentage
No. of patients included	6	7		
Age, years				
Median	52			
Range	(38‐66)			
Sex				
Male	4	66.7	3	42.9
Female	2	33.3	4	57.1
ECOG				
0	1	16.7	3	42.9
1	5	83.3	4	57.1
Smoking history				
Non‐smoker	3	50	6	85.7
Smoker	3	50	1	14.3
EGFR status				
19 del	2	33.3	3	42.9
21 L858R	4	66.7	4	57.1
No. of metastatic organs				
Bone	3	50	6	85.7
Distant Lymph Node	5	83.3	4	57.1
Lung	4	66.7	4	57.1
Pleural	4	66.7	3	42.9
Liver	0	0	2	28.6
Adrenal gland	1	16.7	1	14.3
Brain	1	16.7	3	42.9

Abbreviations: ECOG, Eastern Cooperative Oncology Group; EGFR, epidermal growth factor receptor.

### 3.2 Pharmacokinetics

#### 3.2.1 The concentration time curve and pharmacokinetic parameters

**FIGURE 2:**
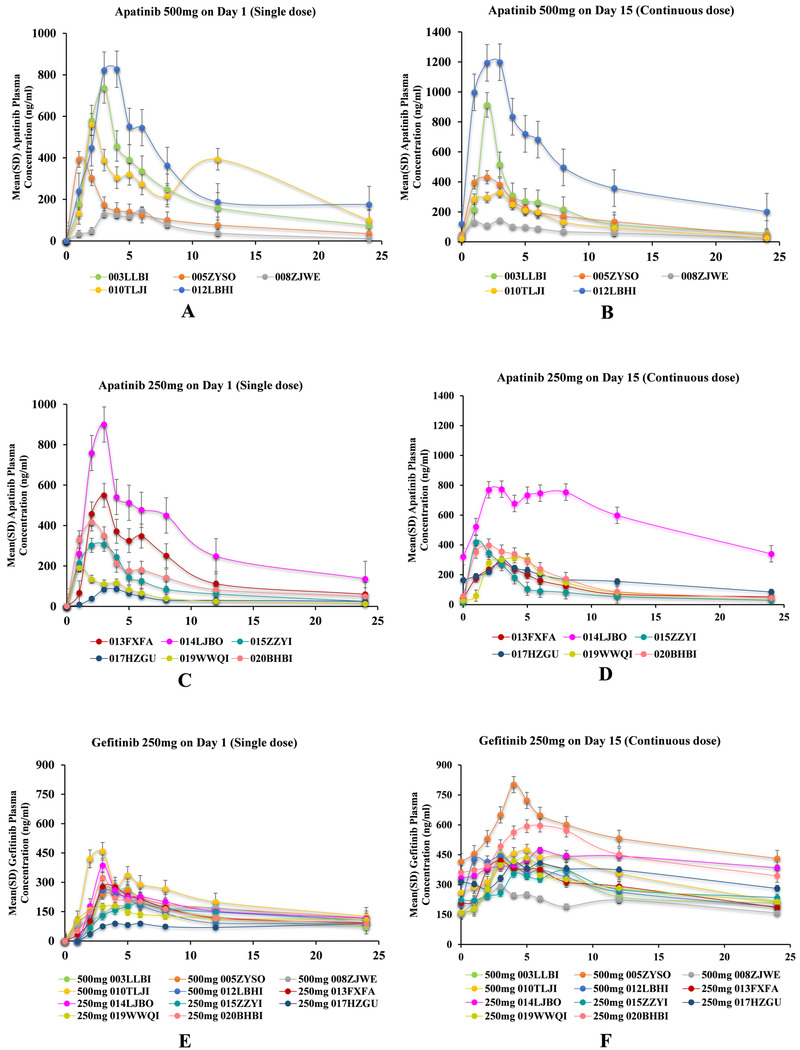
The concentration time curve of apatinib and gefitinib. *A‐D*, Mean Apatinib plasma concentration–time profile on Day 1 (single dose) and Day 15 (continuous dose). Patients were administered with 500 mg or 250 mg Apatinib plus 250 mg Gefitinib once a day; PK evaluation was performed on Cycle 1, Day 1 and Day 15. *E* and *F*, Mean Gefitinib plasma concentration–time profile on Day 1 (single dose) and Day 15 (continuous dose). Patients were administered with 250 mg Gefitinib 250 mg and 500 mg or 250 mg Apatinib once a day; PK evaluation was performed on Cycle 1, Day 1, and Day 15. *Two patients did not have PK data on Cycle 1, Day 15 (one patient had SAE and one was no longer willing to participate and was lost to follow‐up after C1D14)

PK analysis was completed in 11 participants receiving apatinib (500 mg or 250 mg) plus gefitinib (250 mg). The pharmacokinetic parameters of C1, D1 and C1, D15 were calculated using Winnonlin 7 (Table 2). The concentration time curves of apatinib (500 and 250 mg) and gefitinib 250 mg are presented in Figure 2. Detailed pharmacokinetic parameters are listed in Table 2 and Supporting Information.

**TABLE 2:** Estimated PK parameters of apatinib and gefitinib

Dose Level	Apatinib 500 mg		Apatinib 250 mg		Gefitinib 250 mg	
PK parameter	Day 1 (n = 5)	Day 15 (n = 5)	Day 1 (n = 6)	Day 15 (n = 6)	Day 1 (n = 11)	Day 15 (n = 11)
Mean (SD)						
T_1/2_ (h)	10 (3)	9 (2)	17 (20)	11 (3)	21 (14)	30 (17)
Median (range)						
T_max_ (h)	3 (1‐6)	3 (2‐3)	3 (1‐4)	3 (1‐4)	3 (3‐6)	4 (3‐6)
Geometric Mean (%CV)						
C_max_, ng/mL	456 (51)	468 (73)	318 (71)	389 (44)	272 (31)	465 (28)
Geometric Mean (%CV)						
AUC_0‐24_, hr*ng/mL	3807 (57)	3945 (79)	2477 (79)	3898 (91)	3436 (20)	7978 (29)
Geometric Mean (%CV)						
AUC_0‐∞_, hr*ng/mL	4664 (63)	4666 (89)	3507 (70)	4998 (104)	6397 (35)	18101 (62)
Mean (SD)						
Vd (L)	1714 (1006)	1977 (1558)	2154 (2563)	1063 (469)	1100 (275)	1331 (675)
Mean (SD)						
CL (L/h)	144 (133)	1537 (102)	85 (57)	73 (31)	41 (12)	32 (9)

**Abbreviations: T_1/2_**, half‐life; **T_max_,** time to reach C_max_; **C_max_,** maximum plasma concentration; **AUC_0‐24_**, area under plasma concentration‐time curve from 0 to 24 hour; **Vd,** volume of distribution; **CL,** clearance rate; **SD,** standard deviation.

#### 3.2.2 Pharmacokinetic parameters in a steady state

The Css of the apatinib 500 and 250 mg groups were 119.36 ± 108.56 and 101.88 ± 64.62 ng/mL respectively, and the Css of gefitinib was 301 ± 72 ng/mL. The AUCss of the apatinib 500 and 250 mg groups were 2864.73 ± 2605.54 and 2445.09±1550.89 ng mL^–1 ^h^–1^, respectively, and the AUCss of gefitinib was 7224 ±1726 ng mL^–1 ^h^–1^. Other steady‐state concentration PK parameters are shown in Table S5.

### 3.3 Safety

AEs were observed in 13 patients and were similar in both cohorts. The most commonly reported AEs in two cohorts were rash (84.6%, 11/13), diarrhea (69.2%, 9/13), hypertension (69.2%, 9/13), proteinuria (53.8%, 7/13), and hand‐foot syndrome (30.8%, 4/13) (Table 3) and were grades 1–2. One SAE was observed in a patient who had grade 3 hypertension (1/13, 7.7%), which was suspected as treatment‐related (Table S7).

**TABLE 3:** Treatment‐related adverse events (AEs) (n = 13)

	Apatinib 500 mg Cohort (n = 6)			Apatinib 250 mg Cohort (n = 7)		
	ALL	Grade 1–2	Grade 3–4	ALL	Grade 1–2	Grade 3–4
Non‐hematologic AEs						
Skin and subcutaneous tissue disorders						
Rash	6 (100%)	6 (100%)	0	5 (71.4%)	5 (71.4%)	0
Dry skin	1 (16.7%)	1 (16.7%)	0	2 (28.6%)	2 (28.6%)	0
Pruritus	1 (16.7%)	1 (16.7%)	0	0	0	0
Eczema	0	0	0	1 (14.3%)	1 (14.3%)	0
Paronychia	0	0	0	1 (14.3%)	1 (14.3%)	0
HFSR	2 (33.3%)	2 (33.3%)	0	2 (28.6%)	2 (28.6%)	0
Gastrointestinal disorders						
Diarrhea	4 (66.7%)	3 (50.0%)	1 (16.7%)	5 (71.4%)	5 (71.4%)	0
Nausea	3 (50.0%)	3 (50.0%)	0	0	0	0
Vomiting	2 (33.3%)	2 (33.3%)	0	0	0	0
Stomatitis	0	0	0	2 (28.6%)	2 (28.6%)	0
Abdominal distention	0	0	0	2 (28.6%)	2 (28.6%)	0
Gastrointestinal bleeding	0	0	0	1 (14.3%)	1 (14.3%)	0
Hematochezia	0	0	0	1 (14.3%)	1 (14.3%)	0
Hepatobiliary and renal disorders						
Elevated ALT	5 (83.3%)	4 (66.7%)	1 (16.7%)	3 (42.9%)	2 (28.6%)	1 (14.3%)
Elevated AST	3 (50.0%)	3 (50%)	0	2 (28.6%)	1 (14.3%)	1 (14.3%)
Increased bilirubin	1 (16.7%)	1 (16.7%)	0	1 (14.3%)	1 (14.3%)	0
Proteinuria	4 (66.7%)	2 (33.3%)	2 (33.3%)	3 (42.9%)	2 (28.6%)	1 (14.3%)
Increased creatinine	1 (16.7%)	1 (16.7%)	0	0	0	0
Haematuria	0	0	0	1 (14.3%)	1 (14.3%)	0
General disorders						
Hypertension	4 (66.7%)	3 (50.0%)	1 (16.7%)	5 (71.4%)	4 (57.1%)	1 (14.3%)
Fatigue	1 (16.7%)	1 (16.7%)	0	2 (28.6%)	2 (28.6%)	0
Cough	1 (16.7%)	1 (16.7%)	0	4 (57.1%)	4 (57.1%)	0
Dizziness	1 (16.7%)	1 (16.7%)	0	0	0	0
Blurred vision	1 (16.7%)	1 (16.7%)	0	0	0	0
Headache	0	0	0	1 (14.3%)	1 (14.3%)	0
Otorhinolaryngologic disorders						
Epistaxis	1 (16.7%)	1 (16.7%)	0	3 (42.9%)	3 (42.9%)	0
Oulorrhagia	0	0	0	3 (42.9%)	3 (42.9%)	0
Dysgeusia	0	0	0	1 (14.3%)	1 (14.3%)	0
Trachyphonia	1 (16.7%)	1 (16.7%)	0	2 (28.6%)	2 (28.6%)	0
Periodontitis	0	0	0	1 (14.3%)	1 (14.3%)	0
Pharyngalgia	0	0	0	1 (14.3%)	1 (14.3%)	0
Infections and infestations						
Pneumonia (non‐interstitial)	0	0	0	1 (14.3%)	1 (14.3%)	0
Hematologic AEs						
Leukopenia	1 (16.7%)	1 (16.7%)	0	0	0	0
Thrombocytopenia	2 (33.3%)	2 (33.3%)	0	0	0	0
Neutropenia	1 (16.7%)	1 (16.7%)	0	0	0	0

Abbreviations: AST, aspartate transaminase; ALT, alanine transaminase; HFSR, hand foot skin reaction.

### 3.4 Efficacy

**FIGURE 3:**
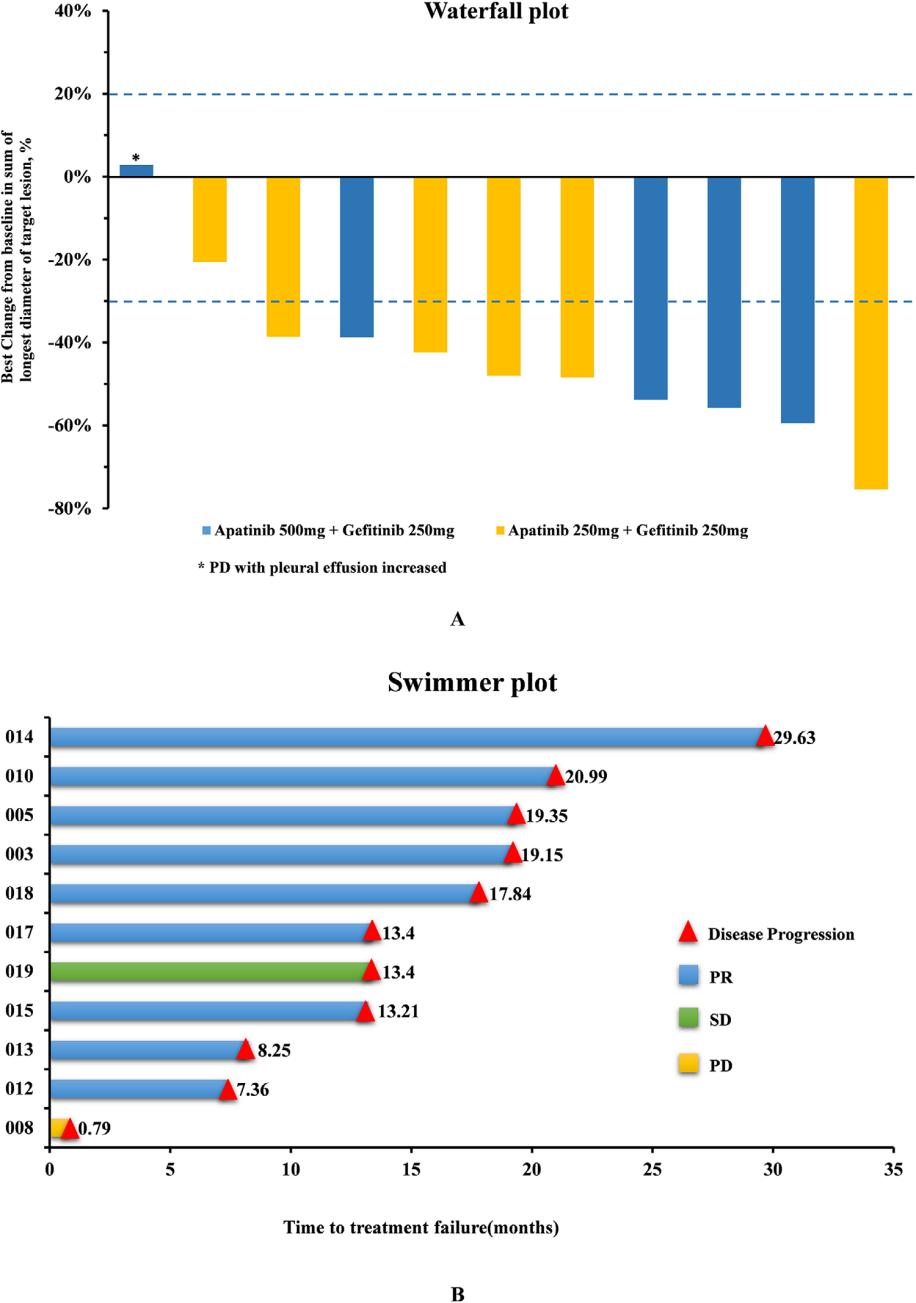
Objective response rate (n = 11). A, Waterfall plot: A maximum tumor change from baseline by best overall response (intention‐to‐treat population). Best change of target‐lesion compared to baseline at best overall response of Apatinib combined with Gefitinib treatment. Waterfall plots for best change of target‐lesion for all patients. The colored bars represent the different doses of Apatinib. The dashed lines are at 20% and −30% represents the boundary for determination of PD and PR, respectively. Asterisks represent PD. B, Swimmer plot: Time to treatment failure from enrollment to PD during treatment with apatinib and gefitinib. Each bar represents one patient's duration exposure of apatinib plus gefitinib, and each color presents the best response of one patient. ^∆^ represent PD

Based on the 11 evaluable patients for efficacy assessment, no CR was achieved. However, nine participants (81.8%) had a confirmed PR, resulting in the ORR of 81.8% (9/11) and the DCR of 90.9% (10/11). One patient (8.3%) had PD (with increased pleural effusion). The objective responses and duration of treatment are presented using waterfall and swimmer plots in Figure 3.

The ORR in patients treated with apatinib 500 mg (80.0%, 4/5]) was similar with apatinib 250 mg (83.3%, 5/6). The DCR was 100% (6/6) in the apatinib 250 mg group compared with the apatinib 500 mg group that was 80.0% (4/5) (Table S8). There was no significant difference in ORR with regards to groups of 500 mg or 250 mg of apatinib, sex, smoking status, ECOG, or EGFR mutation status (Table S9). The shrinkage rates of the target lesions (compared to baseline) in line with the imaging follow‐ups are shown in Figure S1.

By June 4, 2019 data cutoff, 11 patients developed PD and the overall median PFS (mPFS) was 13.4 months. mPFS was compared by taking the Kaplan‐Meier method that was 19.2 months in Cohort A (apatinib 500 mg + gefitinib 250 mg) and 13.4 months in Cohort B (apatinib 250 mg + gefitinib 250 mg), however, no statistical difference was detected (*P *= .966; Figure S2A,B). With a median follow‐up schedule of 29.7 months, the OS was still immature by the data cutoff point (Figure S2C,D). Figure S3 shows a patient with multiple cavitary lesions treated with the apatinib 500 mg + gefitinib 250 mg regimen who achieved the antiangiogenic effect.

### 3.5 Plasma ct‐DNA sequencing

**FIGURE 4:**
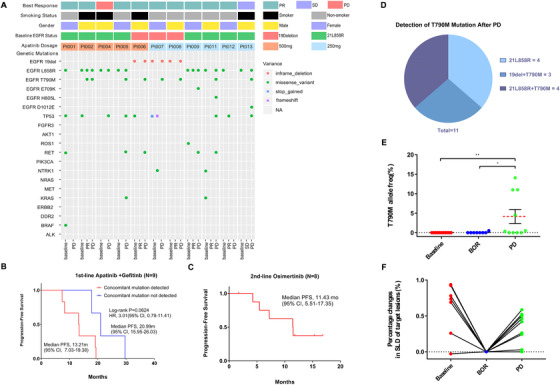
Plasma circulating‐tumor DNA (ct‐DNA) sequencing description summary and clinical outcome exploratory analysis results of 11 patients. A, Mutation plots of sequencing profile at baseline, best of response, and after PD samples, were sorted by the apatinib dosage (500 or 250 mg). Each column represents a distinct patient. BOR, smoking status, and sex groups are shown at the top. B, Kaplan‐Meier curves of progression‐free survival (PFS) in patients whose ct‐DNA had concomitant mutations compared with those without concomitant mutations at baseline. (C) Kaplan‐Meier curves of progression‐free survival (PFS) in 8 patients who received Osimertinib after PD in the second‐line. D, Pie chart depicting the T790M resistant distribution. E, Scatter plot of EGFR‐T790M VAFs (%). The red dashed line represents the median (0 at baseline, 0.6375 at PR/SD, and 4.145 at PD). F, Percentage changes in SLD of target lesions correlated with T790M VAFs. Abbreviations: del, deletion; EGFR, epidermal growth factor receptor; HR, hazard ratio; NR, not reached; PD, progressive disease; PR, partial response; SD, stable disease; PD, progression disease and VAF, variant allele frequency; SLD, sum of the longest diameters

Exploratory analysis was conducted using NGS of plasma ct‐DNA with samples collected at baseline, BOR, and after PD. Patients who acquired resistance to apatinib plus gefitinib (defined as achieving PR, or SD for more than 6 months) were pooled in this analysis.^27^ Eleven participants with available samples were included (BOR: 9 were PR and 1 was SD). The genomic spectrum was displayed in Figure 4A. A concomitant mutation was observed in six out of the nine patients who had samples for NGS detection at baseline. Patients without a concomitant mutation at baseline tended to have a longer PFS when compared with those who had a concomitant mutation (Figure 4B; mPFS, 20.99 months vs 13.21 months; hazard ratio (HR), 3.01 [95%CI, 0.79‐11.41]; *P* = .0624). Of the eight patients who switched to osimertinib, the mPFS reached 11.43 months (Figure 4C). An evaluation of the 11 patients who developed PD status after receiving apatinib plus gefitinib treatment suggests a dominant potential resistance mechanism to the EGFR‐T790M mutation (63.6%, 7/11) (Figure 4D). The ascending EGFR‐T790M variant allele frequency (VAF) after PD was observed in six patients and was significantly correlated with changes in sum of the longest diameters (SLD) of the target lesion (Figure 4E,F). VAF of the T790M at the timepoint of Baseline and BOR were notably lower than that at the timepoint of PD level (median, Baseline vs PD, 0% vs 4.145%, *P* = .0226; BOR vs PD, 0.6375% vs 4.145%, *P* = .0409) (Figure 4F).

## 4 DISCUSSION

To our knowledge, this is the first pilot study that has explored the safety, pharmacokinetic, and efficacy profile of first‐generation EGFR‐TKIs (gefitinib of 250 mg) in combination with an oral VEGFR‐TKIs (apatinib of 500 or 250 mg) for EGFR‐mutant advanced non‐squamous NSCLC. In current study, we found that the regimen of apatinib plus gefitinib exhibited a tolerable safety profile as well as an encouraging antitumor efficacy. Combined with PK analysis, safety, and efficacy profile, our results showed that apatinib 500 mg plus gefitinib 250 mg is a more appropriate dose selection for patients. Exploratory analysis of plasma ct‐DNA by NGS demonstrated that patients with a concomitant mutation at baseline may derive PFS benefits from combinational therapy of apatinib plus gefitinib.

For NSCLC patients treated with gefitinib, resistance usually develops with a median PFS of about 10‐12 months in the first‐line setting.^8^, ^9^, ^10^, ^11^, ^12^ To overcome the resistance developed by EGFR‐TKI monotherapy, the exploration into combinational strategies was put forward, in particular, the combination of an anti‐EGFR plus an anti‐VEGFR. The phase II BELIEF study showed that bevacizumab plus erlotinib exhibited an encouraging prolonged mPFS (13.2 months, 95% CI 10.3‐15.5).^21^ This same regimen was confirmed as effective and tolerable in the JO25567 study^16^ (16.0 months vs 9.7 months, HR 0.54, *P *= .0015), as well as in the phase III NEJ026 study^17^ (16.9 months vs13.3 months, HR 0.61, *P *= .0157). Another phase II study also showed that fruquintinib (4 mg/day) combined with gefitinib (250 mg/day) was tolerable in 26 patients with good therapeutic potency, achieving an ORR of 76.5% and a DCR of 100%.^28^ All these findings highlighted the great potential of an anti‐VEGFR combined with an anti‐EGFR for EGFR‐mutant advanced NSCLC.

Apatinib is a new antiangiogenic small‐molecule oral agent with an acceptable safety profile. Preclinical studies have shown that VEGF‐mediated endothelial cell migration and proliferation can be specifically regulated and inhibited by apatinib.^22^, ^23^, ^24^, ^29^, ^30^ A recent study showed that in mice models of H1975 transplanted tumors, the significant decrease of Ki67 and PCNA expression was observed after the intervention of apatinib plus gefitinib when compared with control group of apatinib or gefitinib alone. This study indicated that adding apatinib to gefitinib may synergistically inhibit the growth of NSCLC and potentially enhance the efficacy of EGFK‐TKIs monotherapy,^30^ thus providing the theoretical basis for the combination of VEGFR‐TKIs with EGFR‐TKIs as a novel approach for treating NSCLC.

In this current study, the ORR was 81.8% (9/11) and DCR was 90.9% (10/11) in all the evaluated patients. The mPFS was 13.4 months, the OS however, was immature with a median follow‐up schedule of 29.7 months. Notably, the apatinib 500 mg group had a longer PFS in comparison with the apatinib 250 mg group, yet the ORRs were similar (PFS, 19.2 months vs 13.4 months, *P *= .966; ORR, 80% vs 83.3%, *P *= 1.000) (Figure S2 and Table S7). In terms of safety, most treatment‐related adverse events (AEs) were reported grade 1–2. Rash, diarrhea, hypertension, proteinuria, elevated ALT/AST, and mild bleeding were the most commonly documented AEs. No significant differences were detected in overall or in grade III‐IV treatment‐related AEs between the two groups (Table 3).

In the pharmacokinetic analysis, increased C_max_ (488 ± 28 ng/mL) and AUC_0‐24_ (3330 ± 852 ng mL^–1 ^h^–1^) of gefitinib was observed compared with previously reported data (Table S6).^31^, ^32^, ^33^ Our data show the main pharmacokinetic parameters of gefitinib in a combinational setting that the steady state clearance rate decreased, indicating that the exposure duration of gefitinib may be enhanced (Table S5). As established, gefitinib has a relatively large therapeutic window (MTD was 750 mg/day),^34^, ^35^ since the recommended dose for gefitinib was 250 mg/day in clinical practice, it is plausible that by increasing the exposure level of gefitinib in NSCLC patients in combination with apatinib that it may improve efficacy. On the other hand, using this anti‐EGFR in combination with an anti‐VEGFR, the C_max_ and AUC_0‐24_ of apatinib were higher in the 500 mg dosage cohort than in the 250 mg dosage cohort (Table 2 and Tables S5 and S6). It has been proven that apatinib has a strong inhibitory effect on CYP3A4, whereas gefitinib is mainly metabolized.^36^ The pharmacokinetic parameters of apatinib in such combined therapy settings revealed that, in contrast, apatinib has a higher accelerated clearance with a reduced AUC (Table S5), which may contribute to the reduction of apatinib‐related toxicity. Taking the pharmacokinetic results into account, the apatinib 500 mg cohort that exhibited a higher C_max_ and AUC_0‐24 h_ of gefitinib appears to be an appropriate dose selection for phase III trials.

It was proven that concomitant mutations were ubiquitous in patients who harbored with EGFR‐mutant advanced NSCLC and that NSCLC may no longer be treated as a single oncogene‐driven disease.^37^, ^38^ Our study also explored the correlation between patients’ concomitant mutation status at baseline and the combination therapy of an EGFR‐TKI plus an VEGFR‐TKI. Nine out of 11 patients had baseline samples for plasma ct‐DNA sequencing and the results showed that a baseline concomitant mutation was detected in 66.7% (6/9) of patients, but remained undetected in 33.3% of patients (3/9). As previously reported, EGFR‐mutant NSCLC patients with detected concomitant mutations at baseline are more likely to have shorter PFS (6.20 months) compared with those without a concomitant mutation (18.77 months).^38^ Our results were similar (Figure 4B, mPFS, 13.21 [95% CI, 7.03‐19.38] months vs 20.99 [95% CI, 15.95‐26.03] months; HR, 3.01 [95% CI, 0.79‐11.41]; *P* = .0624). However, our study showed that a numerically prolonged PFS of 13.21 months in comparison with a historically reported PFS of 6.2 months for patients with a concomitant mutation at baseline,^38^ suggesting that a dual blockade of EGFR and VEGFR may improve the PFS for such patients. Due to the limited sample size and genomic detection information, this hypothesis requires further investigation in future larger‐scale studies. Hopefully, this combination strategy of apatinib plus gefitinib may become a new option for patients harboring the concomitant mutation at baseline. Similar to previously reported results, our analyses showed that the T790M mutation (7/11) remained the leading potential resistance mechanism after PD.^39^, ^40^ Impressively, in the 11 patients who developed PD, eight patients (7 were T790M+ and 1 was T790M‐) switched to second‐line osimertinib and reached a mPFS of 11.43 months (Figure 4C, mPFS, 11.43 [95% CI, 5.51‐17.35] months), which were similar to the AURA3 study results (10.1 months).^41^

Our study has some limitations. First, sample size of our study was comparatively small and by the data cutoff the OS was immature and needed further follow‐up. The post‐study treatment data will be reported in a follow‐up plan with updated OS results. Second, the detection of plasma ct‐DNA for patients was not conducted in all 12 patients as some samples were unavailable or inapplicable for NGS analysis. Therefore, the results of the subgroup PFS analysis should be interpreted with caution. Third, this pilot study was exploratory in design and aimed to observe the preliminary PK and safety profile of this combination therapy. More rigorous drug‐drug interaction (DDI) pharmacological design is needed to better understand the underlying interaction in such combination therapies of EGFR‐TKIs and VEGFR‐TKIs. We have also launched another phase I study focusing on the DDI between apatinib and gefitinib in the hope we can reveal its interactive mechanisms. Recently, two more phase III studies (ARTEMIS and RELAY) whose primary endpoint was PFS, achieved positive results. In the ARTEMIS (CTONG 1509) study, mPFS was 18.0 months for participants treated with erlotinib combined with bevacizumab compared to 11.3 months for participants having erlotinib monotherapy (HR 0.55; *P* < .001).^42^ In the RELAY study, PFS was significantly longer in the experimental arm of erlotinib plus ramucirumab (19.4 months) than in the control arm of erlotinib plus placebo (12.4 months), with a HR of 0.59 (*P *< 0.0001).^43^ These results reveal that an anti‐VEGF combined with an anti‐EGFR could be a ‐new option for NSCLC patients. Based on the encouraging antitumor activity in our current study, we propose that this novel regimen of apatinib plus gefitinib could be another promising combinational first‐line therapy and would be beneficial for patients who were diagnosed with the advanced EGFR‐mutant NSCLC. In order to overcome the aforementioned limitations, we have designed and launched an ongoing multicenter, randomized, placebo‐controlled phase III clinical trial, hoping to further testify the efficacy and safety of apatinib (500 mg) plus gefitinib (250 mg) as a first‐line option for EGFR‐mutant NSCLC (NCT02824458).^18^

## 5 CONCLUSION

Apatinib plus gefitinib shows a manageable tolerability and promising efficacy profile for EGFR‐mutant NSCLC patients as a first‐line treatment. Phase III trials of apatinib (500 mg) plus gefitinib (250 mg) are warranted

## ETHICS APPROVAL AND CONSENT TO PARTICIPATE

This trial has been approved by the institutional review boards (ID: 2016‐FXY‐023) and the independent ethics committees of Sun Yat‐sen University Cancer Center (Ethic approval ID: 5010‐2016‐03). And other participating centers have also obtained the approval from their institutional review boards/independent ethics committees before enrollment. All patients will be required to sign the informed consent form before enrollment. This study was also registered with www.clinicaltrials.gov (ID: NCT02824458).

## CONSENT FOR PUBLICATION

The authors were fully responsible for all content and editorial decisions were involved at all stages of manuscript development, and have approved the final version.

## AVAILABILITY OF DATA AND MATERIALS

The datasets used and/or analyzed during the current study are available from the corresponding author on reasonable request. The data supporting our trial will be found at the online Research Data Deposit website (http://www.researchdata.org.cn) (RDD number: RDDA2019000939)

## CONFLICT OF INTEREST

The authors declare no conflict of interest.

## FUNDING INFORMATION

This work was supported by National Key R&D Program of China (Grant number: 2016YFC0905500, 2016YFC0905503), The Science and Technology Planning Project of Guangdong Province of China (Grant number: 2017B020227001), Natural Science Foundation of Guangdong Province of China (Grant number: 2018A0303130243) and The 5010 Clinical Research Foundation of Sun Yat‐sen University (Grant number: 2016001) and Jiangsu Hengrui Medicine Co. Ltd. (Jiangsu, China). Li Zhang has received research support from the Strategic Priority Research Program of the Chinese Academy of Sciences (No. XDA12020101 to J.D.). Yan Huang has received research support from Science and Technology Program of Guangzhou (201704020072). Yunpeng Yang was supported by Outstanding Young Talents Program of Sun Yat‐sen University Cancer Center (16zxyc03) and Central Basic Scientific Research Fund for Colleges‐Young Teacher Training Program of Sun Yat‐sen University (17ykpy85).

## AUTHOR CONTRIBUTIONS

HZ and LZ made substantial contributions to the design of the study. ZZ, YZ, YM, and FL drafted the manuscript. All investigators planned, coordinated, and conducted the study. ZZ and FL contributed to data management and analysis. ZJ and LS contributed to pharmacokinetic analysis. All authors contributed to the implementation of the study, were involved in revising the manuscript critically, and gave their final approval of the version to be published.

## DISCLOSURES

Presented, in part, at the IASLC 19^th^ World Conference on Lung Cancer (IASLC WCLC 2018) as poster presentation in Advanced NSCLC Session (Abstract Number: P1.01‐109), Toronto, Canada, September 24, 2018

## Supporting information

Supporting information
