# Supplementary material for: Dual blockade of EGFR and VEGFR pathways: Results from a pilot study evaluating apatinib plus gefitinib as a first‐line treatment for advanced EGFR‐mutant non‐small cell lung cancer
Source: Clin Transl Med. 2020 Jun 4;10(2):e33. doi: 10.1002/ctm2.33 (PMC7403827; doi:10.1002/ctm2.33)
Supplement: Supplementary file 1 — Supporting information [file CTM2-10-e33-s001.docx]

**SUPPLEMENTARY MATERIALS**

**Patients and methods**

**Additional Patient Enrollment Criteria**

1) Aged between18~75 years old at enrollment

2) Eastern Cooperative Oncology Group (ECOG) performance status (PS) between 0~1

3) Life expectancy of 12 weeks or more at the time of registration

4) Histologically confirmed diagnosis of stage ⅢB (not suitable for radiotherapy) or Ⅳ non-squamous NSCLC with the presence of measurable tumor lesions (on CT scan according to the Response Evaluation Criteria in Solid Tumors [RECIST] 1.1; tumor lesion no larger than 10 mm, lymph node metastasis no smaller than 15 mm, and no previous locoregional therapy such as radiotherapy or cryotherapy was preformed).

5) Primary NSCLC activated EGFR mutation (exon 19 deletion or L858R point mutation in exon 21) confirmed by validated clinically diagnosed methods; mutation detection was performed prior to the trial in newly diagnosed ⅢB-IV nonsquamous NSCLC patients.

6) No history of chemotherapy or other targeted therapy

*Note: Neoadjuvant chemotherapy was permitted. Patients who underwent adjuvant chemotherapy were ineligible if it was defined as a first-line therapy and relapse had occurred within 6 months since the final day of treatment; adjuvant chemotherapy was permitted provided that relapse had occurred more than 6 months since the final day of treatment.*

7) Prior radiotherapy <25% of the bone marrow was allowed (Cristy and Eckerman 1987); however, prior radiotherapy to the whole pelvis or chest was not permitted. Prior radiotherapy needed to be completed at least 4 weeks before enrollment and related acute toxicities resolved. Local lesions treated with radiotherapy were not included among the measurable tumor lesions, unless significant progress of the local lesions was documented at the time of the last radiotherapy treatment. The following data were required:

- - Blood test results as follows:
    1. ANC≥1.5×10^9^/L
    2. PLT≥100×10^9^/L
    3. Hb≥100g/L
  - Biochemical test:
    1. TBIL<1.5×ULN
    2. ALT and AST<2.5×ULN, or ALT and AST<5×ULN for patients with hepatic metastases
    3. BUN and Cr ≤1×ULN, or CCR ≥50 ml/min (Cockcroft-Gault)

1. Reproductive-age women needed to be using effective contraception or to have a pregnancy test (serum or urine) within 7 days before enrollment of negatively reported results and willing to use a proper contraceptive method throughout the study period and until 8 weeks after the last dose of the study treatment. Men were required to use a proper contraceptive method throughout the study period and until 8 weeks after the last dose of study treatment, or be sterile (for example post-vasectomy).
2. Patients joined the trial based on their personal decision making; all provided written informed consent to partake in treatment and follow-ups.

**Molecular Screening for EGFR**

All patients who underwent EGFR mutation testing before receiving apatinib and gefitinib treatment were eligible for this study. Peripheral blood and tumor tissue were collected from all enrolled subjects. Tumor tissue and blood from patients was sent to a local-lab for detection. The screening method included polymerase chain reaction (PCR) direct sequencing, ARMS, or NGS. Patients were enrolled into one of the two cohorts and were required to be positive for the EGFR 19 deletion or the 21 L858R mutation.

**Study Design and Drug Treatment**

***Apatinib dose modification and discontinuation***

An initial dose of 500mg (2 tablets) or 250mg (1 tablet) of apatinib was administrated once a day. For the 500mg dose, the first-time dose reduction was to 250mg and there were no further reductions thereafter. Apatinib was discontinued if 250mg was unacceptable and was substituted with gefitinib until disease progression or intolerable toxicity. The above modification was applied equally to the 250mg dosage.

***Gefitinib dose modification and discontinuation***

In the event of intolerable diarrhea, proven interstitial lung disease, and the onset of eye symptoms, gefitinib was stopped and appropriate treatment for corresponding adverse events was initiated. Dose modification was not subject to age, weight, sex, race or renal function, or the presence of moderate to severe hepatic impairment caused by hepatic metastases. Gefitinib dose modification and subsequent schedule after occurrence of adverse events were at the discretion of the investigator. If gefitinib was discontinued in patients receiving the gefitinib plus apatinib doublet regimen, the continuation of apatinib depended on the potential benefits and was at the discretion of investigator.

***Drug administration***

Patients began fasting at 22:00 one day before blood collection. Patients were also instructed not to drink fluids 1 hour before and 1 hour after drug administration. Blank blood samples were collected 10 minutes before the drug administration. 2 hours after administration, patients were allowed to consume a normal diet but were instructed not to consume: alcohol, tobacco, coffee and or carbonated drinks during the experiment. Patients were advised to rest and avoid strenuous exercise.

Apatinib and gefitinib tablets were taken orally on an empty stomach in the morning at the same time in with 200ml of warm water. If the two drugs were taken separately, apatinib was taken first. The PK blood collection time was taken according to apatinib.

In the event of a missed dose of apatinib and gefitinib in the course of treatment, it was to be taken as soon as possible unless 12 hours had elapsed since their previous dose. Records should be made indicating the time a missed dose was taken.

***Note:*** *In principle, patients with PD were terminated and withdrawn from the trial, but there were some limitations when applying the evaluation method of cytotoxic chemotherapeutic agents to the therapeutic characteristics of molecular targeted drugs. Although some patients had enlarged tumors after molecular targeted therapy, the internal tissues of the tumors were necrotic. CT scans can show a decrease in the internal density of the tumors, which is generally believed to be beneficial to the patients. This protocol stipulates if the patient is willing to take the drug voluntarily, and if the primary researcher considers that continuing treatment may bring about survival benefits, the patient may continue to participate in the study until the treatment is intolerable or the disease progresses once again. Close observation is required during the process.*

**Pharmacokinetics and Pharmacodynamic Analyses**

***Blood collection administration***

Cycle 1, Day 1 (C1D1): 10 minutes before treatment and 1, 2, 3, 4, 5, 6, 8, 12, 24h after treatment exposure.

Cycle 1, Day 15 (C1D15): 10 minutes before treatment and 1, 2, 3, 4, 5, 6, 8, 12, 24h after treatment exposure.

Collection of minimum concentration: blood samples were collected 30 minutes before treatment exposure on Cycle 1 and Cycle 2, Day 28. The two-day blood collections allowed for a window period of ±2 days.

Unexpected PK blood collection: if SAE occurred, discontinuation or confirmation of PD occurred, blood samples were collected for PK analysis as soon as possible; and no later than 48 hours.

The time window for each blood sample collection: within 10 minutes before the first treatment exposure; window period of ±5 minutes for 1, 2, 3, 4, 5, 6, 8 hours after treatment exposure; window period of ±10 minutes for 12, 24 hours after treatment exposure.

***PK Assessment***

In the two cohorts, PK assessment consisted of a single-dose-administration (C1D1) test and a continuous-dose-administration (C1D15) test. Serial peripheral blood samples were collected at pre-dose and 1, 2, 3, 4, 5, 6, 8, 12 and 24 post-dose for the single dose PK test. For the continuous PK test, serial peripheral blood samples were collected at pre-dose and at 0.5, 1, 2, 3, 4, 5, 6, 8, 12, and 24 hours post-dose on day 28. Samples were centrifuged, and plasma frozen at −80°C refrigerator until further analysis.

Apatinib and Gefitinib plasma concentrations were measured using a validated liquid chromatography–tandem mass spectrometry method with a 0.2 ng/ml lower limit of quantification. PK parameters, including C_max_, AUC, time to reach maximum plasma concentration (T_max_), and t_½_, were calculated using non-compartmental methods with Winnonlin 7 software. Descriptive statistics (mean and standard deviation) were estimated for PK parameters. Median values and ranges were provided for time to reach maximum plasma concentration.

**Next Generation Sequencing of ct-DNA**

***Sample information***

Three time points of blood samples were collected from patients. The initial time point was set at baseline before intervention of treatment, the second time point was set when the patients had the best response according to the efficacy assessment, the third time point was disease progression.

All the patients were recruited at Sun Yat-sen University Cancer Center (SYSUCC) between July 2016 and April 2017. The study was conducted in accordance with the Declaration of Helsinki principles and approved by the ethics committee of SYSUCC (approved ID: 5010-2016-03-01). All subjects provided written informed consent.

***DNA Extraction***

Peripheral blood was collected in Streck BCT® tubes. Plasma was isolated within 72 houses. Streck tubes were centrifugated at 1600g for 10 min at 4℃ and the upper layer of the plasma transferred to 2.0 ml Eppendorf tubes. Eppendorf tubes were then centrifugated at 16,000g for 10 minutes. The supernatants were then transferred to a new Eppendorf tube. cf-DNA was extracted using the QIAamp Circulating Nucleic Acid kit following the manufacturer’s protocol (Valencia, CA). The concentration of DNA was determined using the Qubit 2.0 Fluorimeter (Carlsbad, CA).

***NGS Library Preparation***

30ng to 80ng purified cf-DNA was subjected to end-repairing and A-tailing reaction, and then adaptors with unique molecular index were ligated to the fragments. DNA were amplified by PCR and then captured with probes using the BGI OseqTM -Lung ctDNA kit (BGI, Shenzhen, China)/T panel. The OseqTM -ctDNA/T panel covered 15 genes correlated with lung cancer (Table S1). The captured DNA was amplified by PCR, purified and circularized to generate single strand DNA circles.

***Sequence Data Analysis***

The raw data was first filtered by SOAPnuke-1.5.6 to eliminate low quality reads and adapter contamination. High quality reads were then mapped to the human genome (hg19) using a BWA aligner v0.7.17 and Picard v1.98 to mark PCR duplications. Finally, a Genome Analysis Toolkit (GATK v4.0) was used to execute alignment refinement, somatic mutations calling and filtration.

**Tables**

**Table S1.** The capture region of BGI Oseq^TM^ -Lung-ctDNA kit

| **Gene Symbol** | **Transcript** | **Chromosome** | **Capture region** | **Mutation Type** |
| --- | --- | --- | --- | --- |
| AKT1 | NM_005163.2 | chr14 | partially Exon12 | SNP |
| ALK | NM_004304.4 | chr2 | Exon1-13, Intron10-12 | Fusion, SNP, INDEL |
| BRAF | NM_004333.4 | chr7 | partially Exon4, partially Exon8 | SNP |
| EGFR | NM_005228.4 | chr7 | Exon18-21 | SNP, INDEL |
| ERBB2 | NM_004448.3 | chr17 | Exon20 | SNP |
| FGFR3 | NM_001163213.1 | chr4 | Intron17 | Fusion |
| KRAS | NM_033360.3 | chr12 | partially Exon4, partially Exon5 | SNP |
| MAP2K1 | NM_002755.3 | chr15 | partially Exon2 | SNP |
| MET | NM_000245.3 | chr7 | Exon14, Intron13 | SNP, INDEL |
| NRAS | NM_002524.4 | chr1 | partially Exon5, partially Exon6 | SNP |
| NTRK1 | NM_002529.3 | chr1 | Exon8-12, Intron8-11 | Fusion |
| PIK3CA | NM_006218.3 | chr3 | partially Exon10, partially Exon21 | SNP |
| RET | NM_020975.4 | chr10 | Exon9-19, Intron9-11 | Fusion |
| ROS1 | NM_002944.2 | chr6 | Exon1-13, Intron8-12 | Fusion |
| TP53 | NM_000546.5 | chr17 | Exon2-10 | SNP |

**Table S2.** Details of BGI OseqTM -Lung ctDNA kit

| **Gene list for BGI Oseq (n=15)** | | |
| --- | --- | --- |
| ALK | Fusion | EML4-ALK |
|  |  | KIF5B-ALK |
|  |  | KLC1-ALK |
|  |  | HIP1-ALK |
|  | insertion mutation | ALK_T1151dup |
|  | missense | ALK_G1123S |
|  |  | ALK_L1152R |
|  |  | ALK_C1156Y |
|  |  | ALK_I1171S |
|  |  | ALK_I1171T |
|  |  | ALK_I1171N |
|  |  | ALK_F1174L |
|  |  | ALK_F1174C |
|  |  | ALK_F1174V |
|  |  | ALK_L1196M |
|  |  | ALK_L1198F |
|  |  | ALK_G1202R |
|  |  | ALK_S1206Y |
|  |  | ALK_F1245C |
|  |  | ALK_G1269A |
| BRAF | missense | BRAF_Y472C |
|  |  | BRAF_V600E |
| DDR2 | missense | DDR2_S768R |
| EGFR | missense | EGFR_G719A |
|  |  | EGFR_G719S |
|  |  | EGFR_G719C |
|  |  | EGFR_G719D |
|  |  | EGFR_L747S |
|  |  | EGFR_D761Y |
|  |  | EGFR_S768I |
|  |  | EGFR_T790M |
|  |  | EGFR_C797S |
|  |  | EGFR_T854A |
|  |  | EGFR_L858R |
|  |  | EGFR_L861Q |
|  |  | EGFR_L861R |
|  | Exon 19 deletion | EGFR_I744_A750delinsVK |
|  |  | EGFR_I744_E749delinsLKR |
|  |  | EGFR_E746del |
|  |  | EGFR_E746_L747delinsNY |
|  |  | EGFR_E746_E749del |
|  |  | EGFR_K745_E749del |
|  |  | EGFR_K745_A750del |
|  |  | EGFR_K745_A750delinsT |
|  |  | EGFR_E746_A750delinsIP |
|  |  | EGFR_E746_T751del |
|  |  | EGFR_E746_A750del |
|  |  | EGFR_E746_A750delinsRP |
|  |  | EGFR_E746_A750delinsQP |
|  |  | EGFR_E746_A750delinsAP |
|  |  | EGFR_E746_A750delinsVP |
|  |  | EGFR_E746_A750delinsDP |
|  |  | EGFR_E746_T751delinsA |
|  |  | EGFR_E746_T751delinsS |
|  |  | EGFR_E746_T751delinsI |
|  |  | EGFR_E746_T751delinsIP |
|  |  | EGFR_E746_T751delinsQ |
|  |  | EGFR_E746_T751delinsL |
|  |  | EGFR_E746_T751delinsVA |
|  |  | EGFR_E746_T751delinsVP |
|  |  | EGFR_E746_T751delinsV |
|  |  | EGFR_E746_T751delinsFPS |
|  |  | EGFR_E746_S752delinsA |
|  |  | EGFR_E746_S752delinsV |
|  |  | EGFR_E746_S752delinsD |
|  |  | EGFR_E746_S752delinsI |
|  |  | EGFR_E746_S752del |
|  |  | EGFR_E746_P753delinsLS |
|  |  | EGFR_E746_P753delinsIS |
|  |  | EGFR_E746_P753delinsVS |
|  |  | EGFR_E746_P753delinsVQ |
|  |  | EGFR_L747_P753del |
|  |  | EGFR_L747_R748delinsFP |
|  |  | EGFR_L747_E749del |
|  |  | EGFR_L747_A750delinsP |
|  |  | EGFR_L747_A750delinsS |
|  |  | EGFR_L747_T751delinsP |
|  |  | EGFR_L747_T751del |
|  |  | EGFR_L747_T751delinsS |
|  |  | EGFR_L747_T751delinsQ |
|  |  | EGFR_L747_T751delinsA |
|  |  | EGFR_L747_T751delinsN |
|  |  | EGFR_L747_S752del |
|  |  | EGFR_L747_S752delinsQ |
|  |  | EGFR_L747_S752delinsQH |
|  |  | EGFR_L747_P753delinsQ |
|  |  | EGFR_L747_P753delinsS |
|  |  | EGFR_L747_K754delinsANKG |
|  |  | EGFR_L747_K754del |
|  |  | EGFR_L747_K754delinsST |
|  |  | EGFR_L747_A755delinsAN |
|  |  | EGFR_L747_A755delinsSKG |
|  |  | EGFR_A750_E758del |
|  |  | EGFR_T751_A755del |
|  |  | EGFR_A750_E758delinsP |
|  |  | EGFR_A750_I759delinsPT |
|  |  | EGFR_A750_I759delinsGS |
|  |  | EGFR_T751_I759delinsS |
|  |  | EGFR_T751_E758del |
|  |  | EGFR_T751_I759delinsN |
|  |  | EGFR_T751_I759delinsREA |
|  |  | EGFR_S752_I759del |
|  |  | EGFR_P753_I759del |
|  | EXON 19 insertion | EGFR_I740_K745dup |
|  |  | EGFR_K745_E746insVPVAIK |
|  |  | EGFR_K745_E746insTPVAIK |
|  |  | EGFR_N756dup |
|  | EXON 20 insertion | EGFR_A763_Y764insFQEA |
|  |  | EGFR_A767_S768insIA |
|  |  | EGFR_A767_S768insTLA |
|  |  | EGFR_A767_V769dup |
|  |  | EGFR_V769_D770insGVV |
|  |  | EGFR_V769_D770insGSV |
|  |  | EGFR_V769_N771dup |
|  |  | EGFR_V769_D770insCV |
|  |  | EGFR_V769dup |
|  |  | EGFR_D770_N771insY |
|  |  | EGFR_S768_D770dup |
|  |  | EGFR_D770_N771insGT |
|  |  | EGFR_D770_N771insGL |
|  |  | EGFR_D770_N771insGF |
|  |  | EGFR_D770_N771insGD |
|  |  | EGFR_D770_N771insG |
|  |  | EGFR_D770_N771insAPW |
|  |  | EGFR_D770_N771insT |
|  |  | EGFR_D770_N771insQRG |
|  |  | EGFR_N771_P772insRH |
|  |  | EGFR_N771dup |
|  |  | EGFR_N771_P772insH |
|  |  | EGFR_P772_H773insV |
|  |  | EGFR_P772_H773insTHP |
|  |  | EGFR_H773_V774dup |
|  |  | EGFR_P772_H773insTP |
|  |  | EGFR_H773_V774insQ |
|  |  | EGFR_P772_H773dup |
|  |  | EGFR_N771_H773dup |
|  |  | EGFR_H773dup |
|  |  | EGFR_H773_V774insAH |
|  |  | EGFR_H773_V774insNH |
|  |  | EGFR_V774_C775insPR |
|  | Exon 20 Insertions | EGFR_N771delinsTH |
|  |  | EGFR_N771delinsSH |
|  |  | EGFR_N771delinsSGH |
|  |  | EGFR_D770delinsGY |
|  |  | EGFR_D770_N771delinsAGG |
|  |  | EGFR_N771delinsGY |
|  |  | EGFR_N771delinsGF |
|  |  | EGFR_N771delinsVH |
|  |  | EGFR_N771delinsKL |
|  |  | EGFR_S768_V769delinsIL |
| ERBB2 | EXON 20 insertion | ERBB2_M774delinsWLV |
|  |  | ERBB2_Y772_A775dup |
|  |  | ERBB2_G776delinsLC |
|  |  | ERBB2_G776delinsVC |
|  |  | ERBB2_V777_G778insCG |
|  |  | ERBB2_G778dup |
|  |  | ERBB2_V777_S779dup |
|  |  | ERBB2_G778_P780dup |
|  |  | ERBB2_G778_S779insLPS |
|  |  | ERBB2_G776_V777insL |
| KRAS | missense | KRAS_G12A |
|  |  | KRAS_G12D |
|  |  | KRAS_G12V |
|  |  | KRAS_G12S |
|  |  | KRAS_G12R |
|  |  | KRAS_G12C |
|  |  | KRAS_G12E |
|  |  | KRAS_G12F |
|  |  | KRAS_G12L |
|  |  | KRAS_G12N |
|  |  | KRAS_G12W |
|  |  | KRAS_G12Y |
|  |  | KRAS_G13D |
|  |  | KRAS_G13C |
|  |  | KRAS_G13R |
|  |  | KRAS_G13S |
|  |  | KRAS_G13A |
|  |  | KRAS_G13E |
|  |  | KRAS_G13F |
|  |  | KRAS_G13I |
|  |  | KRAS_G13K |
|  |  | KRAS_G13N |
|  |  | KRAS_G13P |
|  |  | KRAS_G13V |
|  |  | KRAS_G13Y |
|  |  | KRAS_Q61K |
|  |  | KRAS_Q61L |
|  |  | KRAS_Q61R |
|  |  | KRAS_Q61H |
|  |  | KRAS_A146T |
|  |  | KRAS_A146V |
|  |  | KRAS_A146P |
| MET | Exon 14 skipping | MET_c.2888-18_2888-7del12 |
|  |  | MET_c.3024_3028+7del12 |
|  |  | MET_V1001_F1007del |
|  |  | MET_D1010Y |
|  |  | MET_D1010N |
|  |  | MET_D1010H |
|  |  | MET_c.3028+1G>T |
|  |  | MET_c.2888-29_2888-6del24 |
| NRAS | missense | NRAS_G12C |
|  |  | NRAS_G12R |
|  |  | NRAS_G12S |
|  |  | NRAS_G12A |
|  |  | NRAS_G12D |
|  |  | NRAS_Q61K |
|  |  | NRAS_Q61L |
|  |  | NRAS_Q61R |
|  |  | NRAS_Q61H |
| NTRK1 | Fusion | MPRIP-NTRK1 |
|  |  | CD74-NTRK1 |
|  |  | SQSTM1-NTRK1 |
| PIK3CA | Fusion | PIK3CA_H1047R |
|  |  | PIK3CA_H1047L |
| RET | Fusion | KIF5B-RET |
|  |  | CCDC6-RET |
| ROS1 | Fusion | CD74-ROS1 |
|  |  | SDC4-ROS1 |
|  |  | CCDC6-ROS1 |
|  |  | SLC34A2-ROS1 |
|  |  | EZR-ROS1 |
|  |  | TPM3-ROS1 |
|  |  | LRIG3-ROS1 |
|  |  | GOPC-ROS1 |
|  | missense | ROS1_S1986Y |
|  |  | ROS1_S1986F |
|  |  | ROS1_G2032R |
| AKT1 | missense | AKT1_E17K |
| FGFR3 | Fusion | FGFR3-TACC3 |
| TP53 | missense | TP53_S241F |
|  |  | TP53_R273L |

**Results**

***EGFR Mutation Results from Molecular Screening***

**Table S3. EGFR Mutation Status in All Patients before Treatment: Based on Biological Samples**

| EGFR Mutation | Tumor Tissue | Peripheral Blood | Sum |
| --- | --- | --- | --- |
| 19deletion | 4 | 1 | 5 |
| 21L858R | 8 | 0 | 8 |
| Sum | 12 | 1 | 13 |

**Abbreviations:** EGFR, epidermal growth factor receptor

**Table S4. EGFR Mutation Status in All Patients before Treatment: Based on Different Sequencing Methods**

| EGFR Mutation | PCR Sequencing | ARMS | NGS | Sum |
| --- | --- | --- | --- | --- |
| Positive | 1 | 11 | 1 | 13 |

**Abbreviations:** PCR, polymerase chain reaction; ARMS, amplification refractory mutation system; NGS, next generation sequencing.

***Pharmacokinetics***

***The concentration time curve***

PK analysis was performed in 11 patients treated with apatinib (500mg or 250mg) in combination with gefitinib (250mg). Blood samples were collected on the first day of the first cycle (single dose) and the fifteenth day of the first cycle (continuous dose). Blood samples were collected on the twenty-eighth day of the first and the second cycle. The pharmacokinetic parameters of C1, D1 and C1, D15 were calculated using Winnonlin 7 (**Table 2)**. The concentration time curves of apatinib (500mg and 250mg) and gefitinib 250mg are shown in **Figure 2.**

***Pharmacokinetic parameters of single dose and continuous dosages***

The C_max_ of apatinib (500mg dosage level) was 456±273 ng/ml on day 1 and 468±438 ng/ml on day 15, the AUC_0-24h_ was 3807±2616 hr*ng/ml on day 1 and 3945±3898 hr*ng/ml on day 15, and the T_max_ were 3 (1-6) hours and 3 (2-3) hours, respectively. The C_max_ of apatinib (250 mg dosage level) was 318±291 ng/ml on day 1 and 389±184 ng/ml on day 15, the AUC_0-24h_ was 2477±2587 hr*ng/ml on day 1 and 3898±4439 hr*ng/ml on day 15, and the T_max_ were 3(1-4) hours and 3(1-4) hours, respectively. For gefitinib (250 mg fixed dosage), the C_max_ was 272±87ng/ml on day 1 and 465±136 ng/ml on day 15, the AUC_0-24h_ was 3436±702 hr*ng/ml on day 1 and 7978±2390 hr*ng/ml on day 15, and the T_max_ was 3(3-6) hours and 3(3-6) hours, respectively. Detailed PK data are shown in **Table 2.**

**Table S5. Estimated Steady-state PK Parameters of Apatinib and Gefitinib**

| PK parameter | Unit | Mean±SD | | |
| --- | --- | --- | --- | --- |
|  |  | Apatinib 500mg | Apatinib 250mg | Gefitinib 250mg |
| Css | ng/ml | 119.36±108.56 | 101.88± 64.62 | 301±72 |
| AUCss | ng/ml*h | 2864.73±2605.54 | 2445.09±1550.89 | 7224±1726 |
| CLss | L/h | 365.64±305.97 | 127.66±51.39 | 37±10 |
| Vdss | L | 8775.33±7343.36 | 3063.92±1233.25 | 880±234 |

**Abbreviations: Css,** steady state concentration**; AUC_ss_**, area under plasma concentration-time curve for steady state; **Vdss,** steady state volume of distribution**, CLss,** steady state clearance rate**.**

**Table S6.** **Pharmacokinetic Parameters (geometric mean, SD) of Apatinib and Gefitinib**

| PK parameter | Unit | Apatinib 250mg | Apatinib 250mg[1] | Apatinib 500mg | Apatinib 500mg[2] | Gefitinib 250mg | Gefitinib 225mg[3] |
| --- | --- | --- | --- | --- | --- | --- | --- |
| C_max_ | ng/ml | 446 (283) | 581 (321-1216) | 499 (257) | 1521 (75) | 468 (28) | 150 (92) |
| Cmax_multi_ | ng/ml | 415 (184) | NA | 603 (438) | NA | 469 (134) | 341 (61) |
| T_max_ | h | 2.6 (1.0) | NA | 2.8 (1.9) | 3.5 (3-8) | 3.9 (1.2) | 5 (3-7) |
| AUC_0-24_ | ng·h/ml | 3315 (2367) | 3538 (1996-6025) | 4155 (2564) | 11295 (70) | 3330 (852) | 1986 (58) |
| AUC_0-24multi_ | ng·h/ml | 4875 (4439) | NA | 4914 (3898) | NA | 8132 (2311) | 5191 (61) |

**Abbreviations:** Cmax, maximum plasma concentration; Tmax, time to reach Cmax; AUC_0-24_, area under plasma concentration-time curve from 0 to 24 hour; AUC_0-24 multi_, area under plasma concentration-time curve for multiple-dosage regimen

[1]. Yu M, Gao Z, Dai X, et al. Population Pharmacokinetic and Covariate Analysis of Apatinib, an Oral Tyrosine Kinase Inhibitor, in Healthy Volunteers and Patients with Solid Tumors[J]. Clinical Pharmacokinetics, 2017, 56(1):65-76.

[2]. Ding J, Chen X, Dai X, et al. Simultaneous determination of apatinib and its four major metabolites in human plasma using liquid chromatography-tandem mass spectrometry and its application to a pharmacokinetic study[J]. Journal of Chromatography B Analytical Technologies in the Biomedical & Life Sciences, 2012, s 895–896(3):108-115.

[3]. Nakagawa K, Tamura T, Negoro S, et al. Phase I pharmacokinetic trial of the selective oral epidermal growth factor receptor tyrosine kinase inhibitor gefitinib (‘Iressa’, ZD1839) in Japanese patients with solid malignant tumors[J]. Annals of Oncology Official Journal of the European Society for Medical Oncology, 2003, 14(6):922-30.

***Safety***

SAE was observed in one case with grade 3 hypertension (1/13, 7.7%) and was suspected treatment-related **(Table S7)** .Although this patient experienced SAE with hypertension leading to dose interruption, the patient later switched to apatinib 250mg + gefitinib 250mg at SYSUCC’s outpatient clinic and achieved long-term follow-up with no disease progression by the time of the data cutoff.

**Table S7. Serious Adverse Events (n = 1)**

| Patient | Apatinib Dose | Serious Adverse Event | Interruption | Correlation with Apatinib | Ending | Follow-up |
| --- | --- | --- | --- | --- | --- | --- |
| 007 | 500 mg | Hypertension | Discontinuation | Yes | Remission | Received apatinib 250mg + gefitinib 250mg and not yet PD by data cutoff* |

*data cutoff: June, 2019

***Efficacy***

**Table S8. Response Evaluation for Each Dose Cohort (n=11)**

| Apatinib  Dose Cohort | Response in evaluable patient  (n = 11) | | | | Objective Response  n (%) | Disease Control  n (%) |
| --- | --- | --- | --- | --- | --- | --- |
|  | CR | PR | SD | PD | CR+PR | CR+PR+SD |
| 500mg | 0 | 4 | 0 | 1 | 4 (80.0) | 4 (80.0) |
| 250mg | 0 | 5 | 1 | 0 | 5 (83.3) | 6 (100.0) |
| Total | 0 | 9 | 1 | 1 | 9 (81.8) | 10 (90.9) |

**Abbreviations:** CR, complete response; PR, partial response; SD, stable disease; PD, progressive disease

**Table S9. Antitumor Activity of Apatinib in Combination with Gefitinib in Different Patient Subgroups**

| Antitumor Activity | Apatinib Dose Level | | Gender | | Smoking Status | | ECOG | | EGFR Mutation Type | |
| --- | --- | --- | --- | --- | --- | --- | --- | --- | --- | --- |
|  | 500mg | 250mg | Male | Female | Yes | No | 0 | 1 | 19Deletion | 21L858R |
| Responded (%) | 4 (80.0) | 5 (83.3) | 4 (44.4) | 5 (55.6) | 2 (22.2) | 7 (77.8) | 3 (33.3) | 6 (66.7) | 3 (33.3) | 6 (66.7) |
| Not responded | 1 | 1 | 2 | 0 | 2 | 0 | 0 | 2 | 0 | 2 |
| Sum | 5 | 7 | 7 | 5 | 4 | 8 | 3 | 9 | 4 | 8 |
| *P* value | 1.000 ^a^ | | 0.455^a^ | | 0.109 ^a^ | | 1.000 ^a^ | | 1.000 ^a^ | |

^a^ Fisher's Exact Test

**Abbreviations:** ECOG, Eastern Cooperative Oncology Group; EGFR, epidermal growth factor receptor

| No. | Initials | Age | Gender | EGFR mutation | ECOG | Smoking | BOR | 1st-line Treatment | 1st-line PFS  (m) | 1st-line PD status | 2nd-line Treatment | 2nd-line PFS  (m) | 2nd-line PD status | Death Status | OS  (m) |
| --- | --- | --- | --- | --- | --- | --- | --- | --- | --- | --- | --- | --- | --- | --- | --- |
| 001 | LLBI | 52 | Female | 21L858R | 0 | Non-smoker | PR | Apatinib 500mg  +Gefitinib 250mg | 19.15 | PD | Osimertinib | 17 | Non-PD | Alive | 34.79 |
| 002 | ZYSO | 53 | Male | 21L858R | 1 | Smoker | PR | Apatinib 500mg  +Gefitinib 250mg | 19.35 | PD | NA | NA | NA | Death | 22.18 |
| 004 | ZJWE | 47 | Male | 21L858R | 1 | Smoker | PD | Apatinib 500mg  +Gefitinib 250mg | 0.79 | PD | Pemetrexed+Carboplatin+ Bevacizumab→Pemetrexed+Bevacizumab Maintenance | 11.5 | PD | Alive | 33.58 |
| 005 | TLJI | 56 | Female | 21L858R | 1 | Non-smoker | PR | Apatinib 500mg  +Gefitinib 250mg | 20.99 | PD | Osimertinib | 12 | Non-PD | Alive | 31.87 |
| 006 | LBHA | 60 | Male | 19del | 1 | Smoker | PR | Apatinib 500mg  +Gefitinib 250mg | 7.36 | PD | Osimertinib | 5.5 | PD | Death | 26.25 |
| 007 | FXFA | 38 | Female | 19del | 1 | Non-smoker | PR | Apatinib 250mg  +Gefitinib 250mg | 8.25 | PD | Osimertinib | 11.5 | PD | Alive | 29.7 |
| 008 | LJBO | 48 | Male | 19del | 0 | Non-smoker | PR | Apatinib 250mg  +Gefitinib 250mg | 29.63 | PD | Osimertinib | 1.7 | Non-PD | Alive | 29.63 |
| 009 | ZZYI | 40 | Female | 21L858R | 0 | Non-smoker | PR | Apatinib 250mg  +Gefitinib 250mg | 13.21 | PD | Osimertinib | 4.3 | PD | Alive | 30.39 |
| 011 | HZGU | 66 | Male | 21L858R | 1 | Non-smoker | PR | Apatinib 250mg  +Gefitinib 250mg | 13.4 | PD | Osimertinib | 15.6 | Non-PD | Alive | 29.34 |
| 012 | WWQI | 64 | Female | 21L858R | 1 | Non-smoker | PR | Apatinib 250mg  +Gefitinib 250mg | 17.84 | PD | NA | NA | NA | Death | 24.41 |
| 013 | BHBI | 44 | Male | 21L858R | 1 | Smoker | SD | Apatinib 250mg  +Gefitinib 250mg | 13.4 | PD | Osimertinib | 7.1 | PD | Alive | 27.4 |

**Table S10. Summary** **characteristics and outcomes of 11 patients in FAS**

**Abbreviations:** ECOG, Eastern Cooperative Oncology Group; EGFR, epidermal growth factor receptor; PFS, progression-free survival; OS, overall survival; PD, progressive disease

***Efficacy***

**
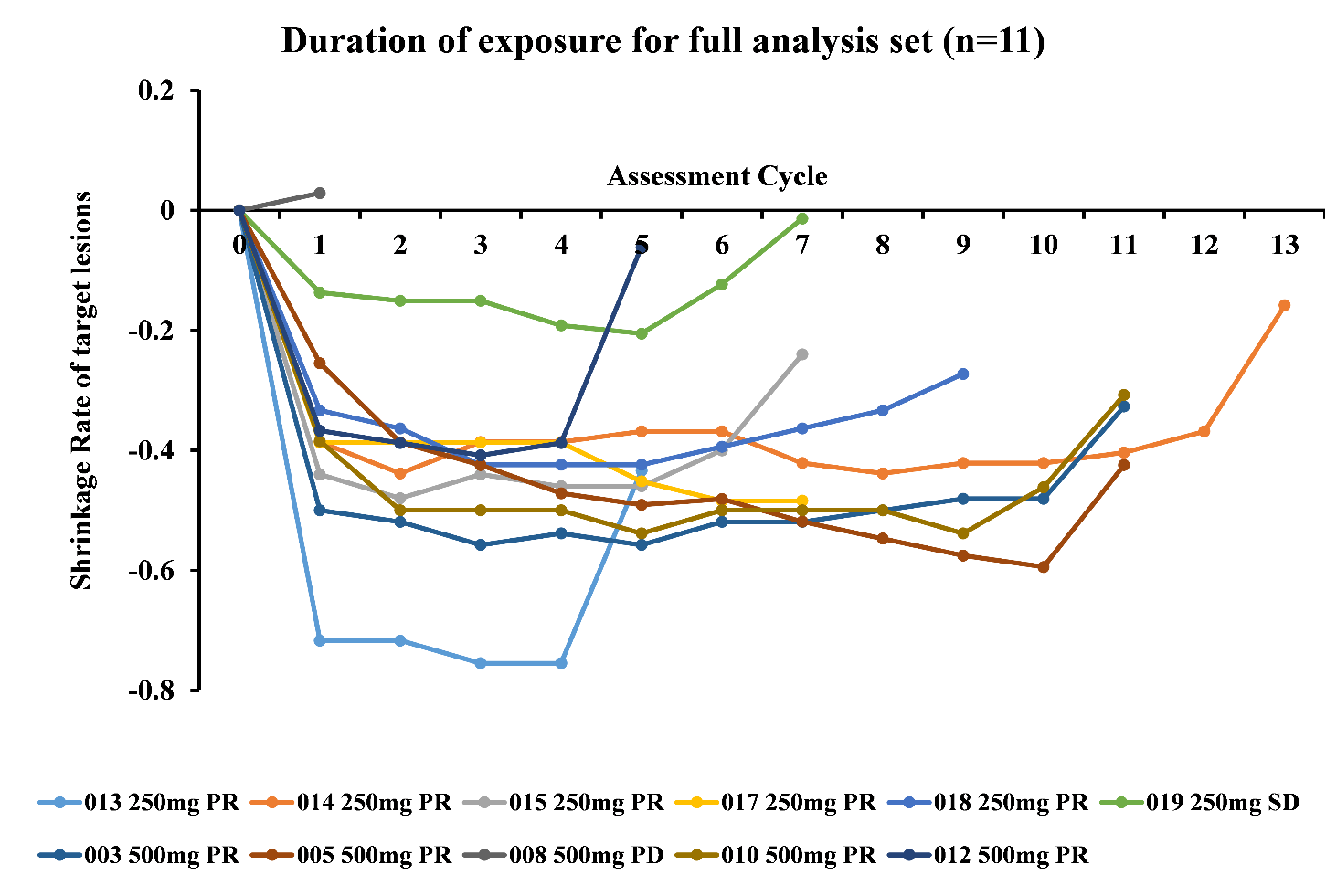
**

**Figure S1. Target lesion size shrinkage rate compared with baseline at each evaluation point during apatinib in combination with gefitinib exposure.** Each line represents one patient’s duration of apatinib + gefitinib exposure; while, each dot represents the change rate compared to baseline at each imaging assessment point. The best response and apatinib dose are listed in the legend.

***
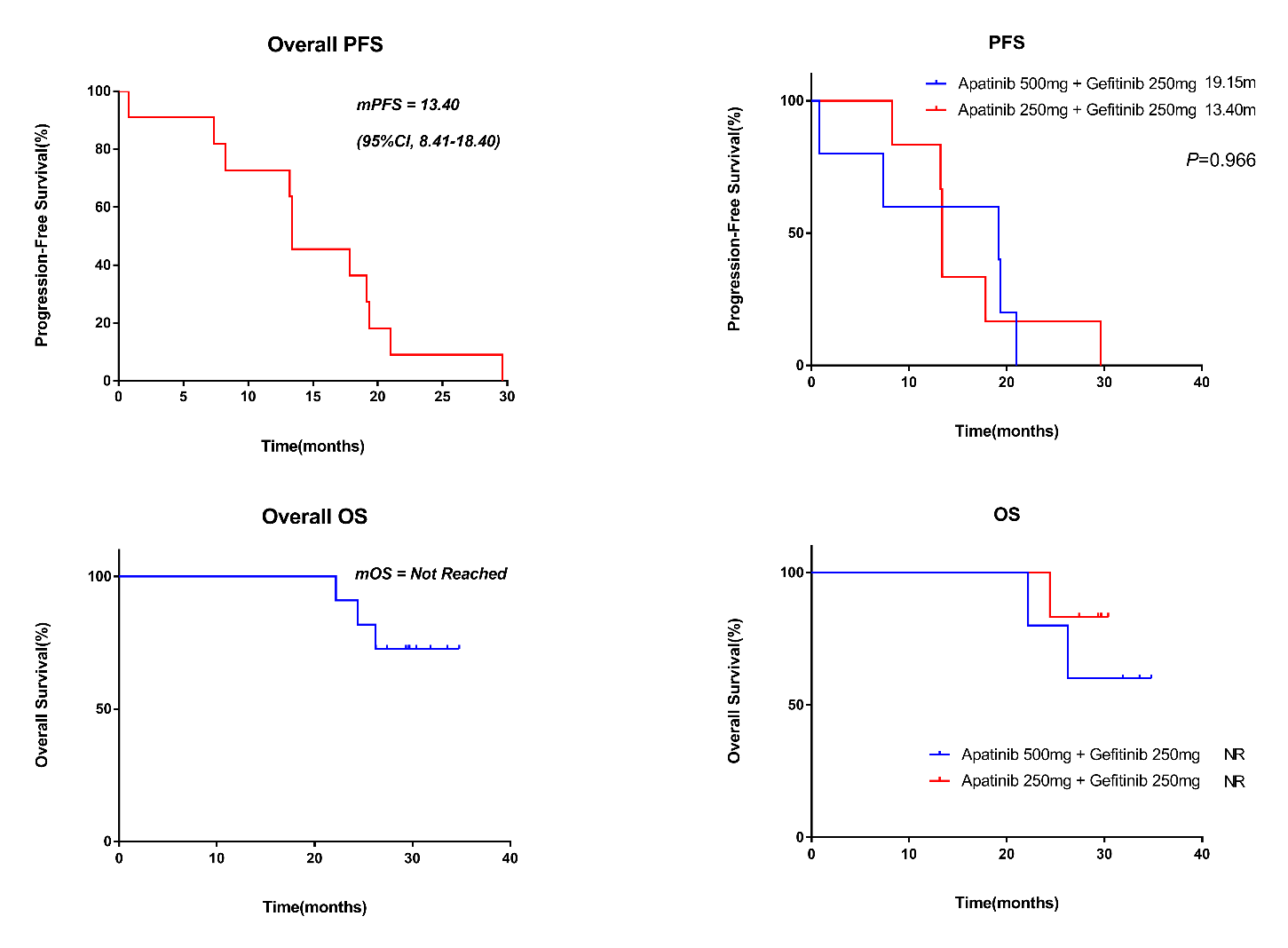
***

**D**

**C**

**A**

**B**

**Figure S2. Progression-free Survival and Overall Survival. (A)** Overall progression-free survival in 11 patients; **(B)** Comparison of progression-free survival in the two groups; **(C)** Overall survival in 11 patients; **(D)** Comparison of overall survival in the two groups.

**
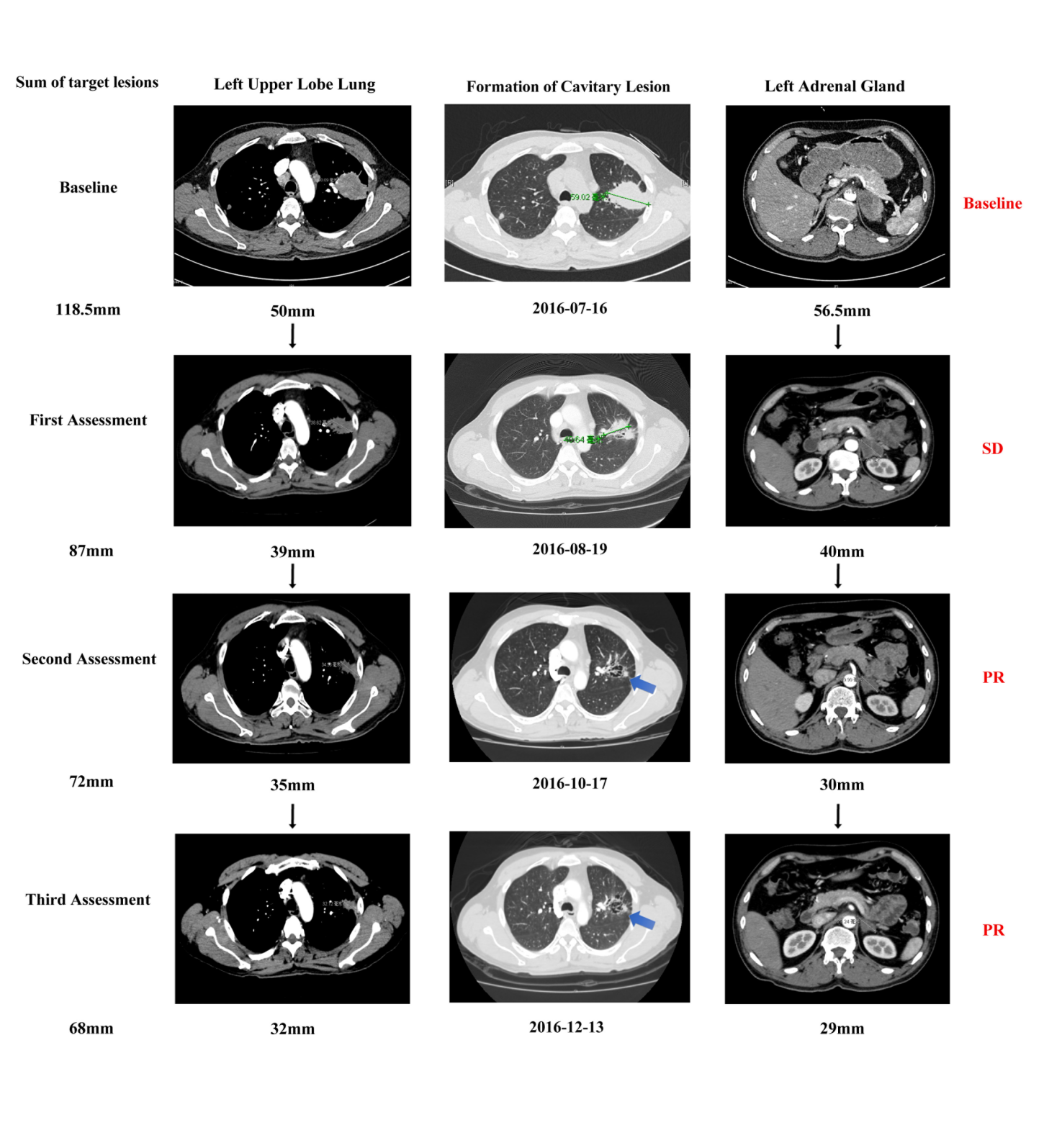
**

**Figure S3. A single** **case of antiangiogenic effects with apatinib (n = 1).** A 53-year-old Chinese male diagnosed in 2016 with a left upper lobe lung adenocarcinoma with pleura, adrenal gland, bone, and brain metastasis, harboring an EGFR 21L858R point mutation. After treating the patient with apatinib 500mg plus gefitinib for 20 weeks the patient achieved a stable disease (SD), then a partial response (PR) based on a regular CT scan assessment. The antiangiogenic effect of apatinib is shown in the CT scan with the formation of multiple cavitary lesions where the lung targeted lesion was assessed.
